# Supplementary material for: Active choice judgement bias reflects affective indicators in bonobos
Source: Anim Cogn. 2026 Jul 19;29(1):74. doi: 10.1007/s10071-026-02084-y (PMC13710652; doi:10.1007/s10071-026-02084-y)
Supplement: Supplementary file 1 [file 10071_2026_2084_MOESM1_ESM.docx]

**Supplementary Figure 1:** Diagram of the bonobo habitats at Twycross Zoo at the time of testing. Red dots indicate the position of the researcher during judgement bias training and testing sessions.
