# Supplementary material for: Active choice judgement bias reflects affective indicators in bonobos
Source: Anim Cogn. 2026 Jul 19;29(1):74. doi: 10.1007/s10071-026-02084-y (PMC13710652; doi:10.1007/s10071-026-02084-y)
Supplement: Supplementary file 2 [file 10071_2026_2084_MOESM2_ESM.docx]

| Authors | Year | Paradigm | Discrimination | Common name | Scientific name | Setting |
| --- | --- | --- | --- | --- | --- | --- |
| Bethell et al | 2012 | Go/ no-go | Visual | Rhesus macaques | *Macaca mulatta* | Laboratory |
| Pomerantz et al | 2012 | Active choice | Visual | Tufted capuchins | *Cebus apella* | Laboratory |
| Bateson and Nettle | 2015 | Go/ no-go | Visual | Chimpanzee | *Pan troglodytes* | Sanctuary |
| Gordon and Rogers | 2015 | Go/ no-go | Visual | Common marmoset | *Callithris jacchus* | Laboratory |
| Ash and Buchanan-Smith | 2016 | Go/ no-go | Visual | Common marmoset | *Callithris jacchus* | Laboratory |
| Schino et al | 2016 | Active choice | Visual | Tufted capuchins | *Cebus apella* | Laboratory |
| Perdue | 2017 | Active choice | Visual | Rhesus macaque and Capuchin | *Macaca mulatta*  *Cebus apella* | Laboratory |
| Adriaense | 2021 | Hybrid | Spatial | Common marmoset | *Callithris jacchus* | Laboratory |
| Iki and Adachi | 2023 | Go/ no-go | Visual | Japanese macaques | *Macaca fuscata* | Laboratory |
| Perea-Rodriguez | 2024 | Go/ no-go | Spatial | Grey Mouse Lemurs | *Microcebus murinus* | Laboratory |
| Winkler et al | 2025 | Go/ no-go | Visual | Bonobo | *Pan paniscus* | Laboratory |
| Iki and Adachi | 2025 | Go/no-go | Visual | Japanese macaques | *Macaca fuscata* | Laboratory |

**Supplementary Table 1**: Complete list of previous judgement bias tasks conducted on nonhuman primates, accurate to the time of publication.
