# Supplementary material for: Active choice judgement bias reflects affective indicators in bonobos
Source: Anim Cogn. 2026 Jul 19;29(1):74. doi: 10.1007/s10071-026-02084-y (PMC13710652; doi:10.1007/s10071-026-02084-y)
Supplement: Supplementary file 3 [file 10071_2026_2084_MOESM3_ESM.docx]

**Supplementary table 2** ethogram used in behavioral observations. Behavior was recorded on the 30 second mark for the focal individual.

| Behavioral category | Behavior code | Description | Modifiers |
| --- | --- | --- | --- |
| Abnormal | Regurgitate & reingest | Animal brings up previously masticated food and reingests; may use fingers to manually gag or rock back and forth. |  |
|  | Urophagy / coprophagy | Animal deliberately ingests feces/ urine from self or conspecific, includes instances when the subject “paints” with feces on an exhibit surface. |  |
|  | Abnormal movement/ posturing | Animal performs repeated, sustained, and/ or purposeless manipulation of a specific area of own body, such as ear/ eye-poking, chest-patting, nipple-pulling or ear-covering. Includes lip-pursing during repetitive vocalizations. |  |
|  | Plucking | Animal deliberately and purposely pulls out own hair using hands and/or mouth. |  |
| Affiliative | Social groom | (Give) Animal picks through hair or skin of conspecific and removes debris with hands and/or mouth. (Receive) Conspecific picks through hair or skin of focal animal and removes debris with hands and/or mouth. | Give  Mutual  Receive |
|  | Social play | Non-aggressive interactions involving two or more conspecifics. Never accompanied by pilo-erection or agonism; may be accompanied by play-face and/or laughing. Includes rough-and-tumble play (fast-paced, vigorous locomotion, wrestling, hitting, pulling, chasing, biting, etc.), quiet play (slower-paced, gentle-tickling, finger and toe manipulation, etc.). May involve the use of an object. May also occur with keeper/ guest and include tickling, chasing, or stomping. |  |
| Agonistic | Contact aggression | (Give) Animal displays an aggressive behavior directed at a conspecific. (Receive) animals receives aggressive behavior from a conspecific. Must involve some physical contact between individuals, includes, wrestling, lunge hit, grab, bite, and scratch. May include pilo-erection. | Give  Receive |
|  | Non-contact aggression | (Give) Animal performs an aggressive behavior directed to a conspecific/ human. (Receive) Animal receives an aggressive behavior from a conspecific. Must not include any physical contact, includes lunge, rush, threats, hitting/ kicking a barrier and stabbing with fingers/ stick. | Give  Receive |
| Other | Other | Animal performs a behavior not otherwise listed. |  |
|  | Out of view | Animal is not in view of camera. |  |
| Sexual | Masturbation | Animal actively uses a body part, object, or part of the cage to stimulate own genitals. |  |
|  | Present | Animal presents stomach with arms open to conspecific, often followed by social sex or genital-genital rub. |  |
|  | Genital-genital rub | Animal presses and rubs own genitals against the genitals of conspecific. May occur in any position or movement pattern except thrusting (score social sex). |  |
|  | Social sex | Animal performs any component of a series of sexual behaviors including mounting and complete copulation - characterized by thrusting with or without penetration. |  |
| Solitary | Auto-groom | Picking through or rubbing own hair or skin and removing debris with hand, foot, object and/or mouth. Does not include plucking hair or rough-scratching. |  |
|  | Explore | Animal touches, manipulates, licks, and sniffs the surrounding environment and non-edible enrichment in a manner too slow and deliberate for play; does not include much locomotion |  |
|  | Feed/ forage/ drink | Animal masticates, collects, and/ or searches for food while in locomotion or extracts food from enrichment with some degree of difficulty. Include pulling grass from the ground, bark stripping, etc. Also includes drinking from tap and suckling. |  |
|  | Inactive | (Alert) Animal is sedentary, sitting up, or laying down with eyes open; head is orientated so that it is looking around at its surroundings. (Resting) Animal is sedentary and positioned in a manner that does not require energy to maintain. Eyes are often closed or blinking, head may be orientated in such a way where it cannot view its surroundings. | Alert  Resting |
|  | Locomote | Animal is moving horizontally or vertically throughout its exhibit - can be climbing, walking, or brachiating throughout the habitat. Includes moving throughout the habitat while holding enrichment, nesting material, or food. |  |
|  | Nesting | Animal manipulates, arranges or rearranges woodwool, branches, leaves etc. in the form of a nest around itself. |  |
|  | Peer | Animal places face/ eyes within a few inches of the face of window/ slide. May be interacting with guests or watching other troop. |  |
|  | Solitary play | Individual may play by itself with hands, fingers and toes, other body parts, or use an object as the focus of or tool for play. Typically includes playful, vigorous and exaggerated movements with no clear purpose. May be either boisterous or quiet (spinning, somersaulting, vigorous shaking of blankets). |  |

**Supplementary table 3** proximity ethogram used in behavior observations. Proximity level from the focal animal was recorded on the 30 second mark for every conspecific in the social group of the focal animal.

| Proximity level | Definition |
| --- | --- |
| Contact | Animal is physically touching a conspecific |
| Far | Animal is outside of an arm's length from a conspecific |
| Near | Animal is within an arm's length of a conspecific |
| Unknown | Proximity to focal cannot be determined |
