# Supplementary material for: Active choice judgement bias reflects affective indicators in bonobos
Source: Anim Cogn. 2026 Jul 19;29(1):74. doi: 10.1007/s10071-026-02084-y (PMC13710652; doi:10.1007/s10071-026-02084-y)
Supplement: Supplementary file 4 [file 10071_2026_2084_MOESM4_ESM.docx]

**Keeper Survey**

AnonID: Date:

Focal animal:

**Qualitative Behavioural Assessment**

Please mark the value that best describes your perception of how the focal animal is feeling today based on your interactions with him/her.

**Content:**

The animal is at ease, tranquil, satisfied

5

4

3

2

1

Content

Not content

**Relaxed:**

The animal appears peaceful and free from tension.

5

4

3

2

1

Relaxed

Not relaxed

**Uncomfortable:**

The animal is ill at ease without a clear context for any distress. Body language is un-relaxed and possibly changing frequently.

5

4

3

2

1

Uncomfortable

Comfortable

**Agitated:**

The animal appears to be in a state of uncertainty which can be accompanied by physical restlessness and over-reaction to stimuli. May be scanning the environment in a tense and anxious fashion.

5

4

3

2

1

Agitated

Not agitated

**Tense:**

The animal is holding itself in a rigid fashion and displays un-relaxed reactions to stimuli.

5

4

3

2

1

Tense

Not tense

**Frustrated:**

The animal is seeking a goal without success; can be violent towards others or objects or the environment and displays an angry body posture.

5

4

3

2

1

Frustrated

Not frustrated

**Wary:**

The animal appears nervous, displays paused reaction to some stimuli or is unwilling to move in or out of an area. This may be accompanied by listening and smelling. Animal is displaying slow and calm behaviour.

5

4

3

2

1

Wary

Not wary

**Playful:**

The animal has engaged in a bout of object, locomotory or social play and is responding positively to solicitations for play.

5

4

3

2

1

Playful

Not playful

*QBA questions modified from Yon, L., Williams, E., Harvey, N. D., & Asher, L. (2019). Development of a behavioural welfare assessment tool for routine use with captive elephants. PLoS One, 14(2), e0210783.*
