# Supplementary material for: Active choice judgement bias reflects affective indicators in bonobos
Source: Anim Cogn. 2026 Jul 19;29(1):74. doi: 10.1007/s10071-026-02084-y (PMC13710652; doi:10.1007/s10071-026-02084-y)
Supplement: Supplementary file 5 [file 10071_2026_2084_MOESM5_ESM.docx]

**Preference testing**

Preference testing trials were conducted to determine which food item each subject preferred, thereby identifying the more positive and less positive rewards. Three non-consecutive preference testing sessions were conducted within the month of March, 2024. Each animal had the opportunity to participate in 10 preference testing trials over the course of each session, combining to a total of 30 possible preference testing trials per individual. During each trial, a one cm^3^ of sweet potato and a single redskin peanut were placed approximately 45 cm apart from each other on a small camping table (Figure 1). The side each item was placed on was randomized to prevent side-bias from confounding the results. The experimenter initiated each trial when an animal calmly approached the testing apparatus and conspecifics seemed unlikely to interfere. The experimenter then pushed the table towards the animal. Animals indicated their preference by sticking their fingers through the mesh at the food item. After a choice was made, the experimenter withdrew the table and placed the food item into a tube that allowed it to fall into the habitat. Occasionally, animals were able to knock a food item off the table or grab it themselves, in which case their choice was noted as for a normal choice.

Every individual in the troop participated in at least 5 preference testing trials over the 3 sessions, with 13 individuals completing more than 10. The individual who only completed 5 trials was excluded from the dataset, as 5 trials were considered too few to indicate a meaningful preference. The rest of the troop had their preferences calculated as the number of trials where the peanut was chosen / all trials completed (Table 2). A score greater than 60% was considered a peanut preference while a score less than 40% was considered a sweet potato preference. Animals who chose peanuts in 40-60% of trials were considered as having no preference. The majority of animals displayed a clear preference for the peanut (Table 1). Preference scores indicating peanut preference ranged from 63-97%. Two individuals, notably a mother and her two-year-old infant, displayed a sweet potato preference. Two more individuals displayed no preference.

**Figure 1:** Preference testing setup used to determine More Positive and Less Positive rewards. AI (ChatGPT-5) was used to create photorealistic images of food rewards.

**Table 1**: Preference testing results for all 13 bonobos who completed more than 5 trials. Individuals with an asterisk next to their name were the same animals who passed learning criteria on the judgement bias test.

| Individual | Sex | Age in years | Matriline information | Total trials | Percent of preference | Final preference |
| --- | --- | --- | --- | --- | --- | --- |
| Bonobo A* | F | 9 | Unrelated to rest of troop | 30 | 0.83 | Peanut |
| Bonobo B | F | 28 | Mother to Bonobos J and L | 30 | 0.30 | Sweet potato |
| Bonobo C | M | 30 | Son of Bonobo N | 17 | 0.59 | None |
| Bonobo D | F | 39 | Mother to Bonobos G, H, and K | 30 | 0.47 | None |
| Bonobo E* | F | 14 | Mother to Bonobo F | 30 | 0.97 | Peanut |
| Bonobo F* | F | 4 | Daughter of Bonobo E | 29 | 0.83 | Peanut |
| Bonobo G | M | 26 | Son of Bonobo D | 10 | 0.90 | Peanut |
| Bonobo H* | M | 21 | Son of Bonobo D | 30 | 0.93 | Peanut |
| Bonobo I | F | 26 | Unrelated to rest of troop | 12 | 0.92 | Peanut |
| Bonobo J* | M | 8 | Son of Bonobo B | 30 | 0.63 | Peanut |
| Bonobo K* | M | 8 | Son of Bonobo D | 30 | 0.63 | Peanut |
| Bonobo L | M | 2 | Son of Bonobo B | 11 | 0.36 | Sweet potato |
| Bonobo M* | F | 9 | Unrelated to rest of troop | 30 | 0.70 | Peanut |
| Bonobo N | F | 46 | Mother of Bonobo C | 5 | N/A | N/A |
